# Remarkable response to PD-1 inhibitor in a patient with extensive-stage small cell lung cancer: a case report and literature review

**DOI:** 10.3389/fimmu.2023.1267606

**Published:** 2023-09-14

**Authors:** Ge Yuan, Xiangliang Liu, Xinwei Zhang, Wei Song, Jin Lu, Zhongyang Ding, Xiao Chen

**Affiliations:** Cancer Center of The First Hospital of Jilin University, Changchun, China

**Keywords:** small cell lung cancer, immune checkpoint inhibitors, PD-1, NLR, CEA

## Abstract

We report a case of a 59-year-old male diagnosed with extensive-stage small cell lung cancer (SCLC). He received first-line platinum doublet chemotherapy and second-line topotecan-based regimen, but experienced disease progression after each line of therapy. He was then treated with Sintilimab, a PD-1 inhibitor, in combination with nab-paclitaxel in the third-line setting, which resulted in significant tumor shrinkage. Restaging scans showed a partial response per RECIST criteria with 62% reduction in tumor burden. This case highlights the application and efficacy of immune checkpoint inhibitors in extensive-stage SCLC.

## Introduction

Small cell lung cancer (SCLC) accounts for approximately 15% of all lung cancer cases and is characterized by rapid doubling time, early metastasis, and poor prognosis (1). Most patients present with metastatic disease at diagnosis, and only one-third are diagnosed at limited stage, where curative multimodality therapy can be delivered (2).

First-line treatment for extensive-stage SCLC consists of platinum doublets (etoposide/cisplatin or irinotecan/cisplatin), but nearly all patients experience disease progression within months of completing chemotherapy (3, 4). Second-line topotecan has limited efficacy and substantial toxicity (5). In recent years, immune checkpoint inhibitors (ICIs) targeting PD-1/PD-L1 have shown promising clinical activity in SCLC and have been approved for first-line and later-line treatment (6, 7). However, only a small subset of SCLC patients respond to ICIs, partly due to lack of tumor-infiltrating lymphocytes (TILs) (8). Here, we present a case where Sintilimab treatment in the third-line setting led to substantial tumor regression and prolonged disease control in an extensive-stage SCLC patient. This exceptional response highlights the potential for ICIs to improve outcomes for selected SCLC patients. Reliable predictive biomarkers are urgently needed to identify SCLC patients most likely to respond to ICIs (9).

## Case presentation

**Figure 1:**
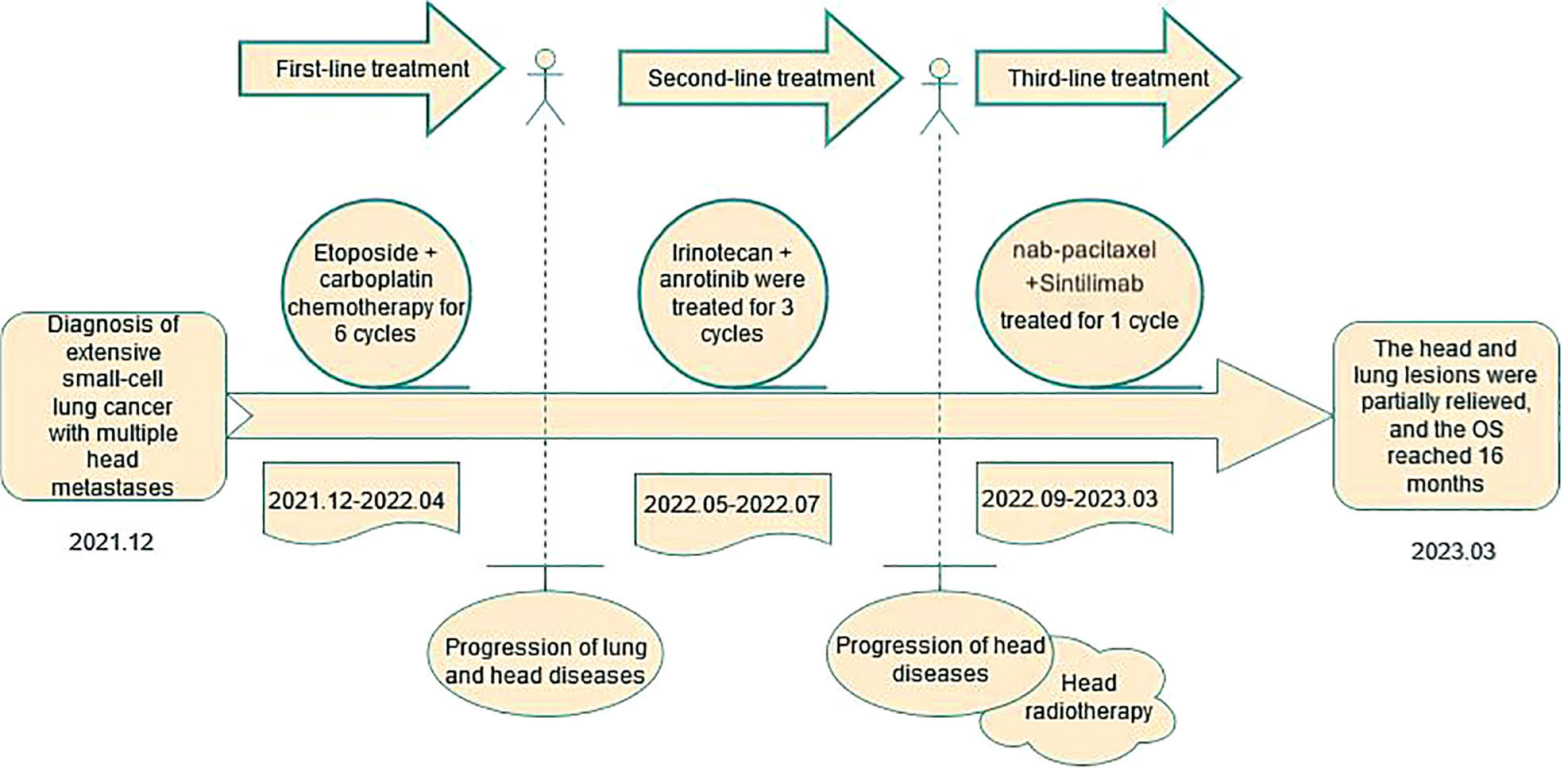
Treatment timeline.

A 59-year-old male, never-smoker, presented with cough and dyspnea in December 2021. Diagnostic workup revealed extensive-stage SCLC with metastases to lymph nodes and brain. He received first-line etoposide/cisplatin for 6 cycles with best response of PR. In May 2022, restaging CT showed disease progression in the lungs and lymph nodes. Second-line irinotecan/anlotinib was administered for 3 cycles but the patient experienced further progression in the brain. He underwent palliative brain radiation in July 2022. In September 2022, the lung lesions enlarged again on CT. Third-line treatment with nab-paclitaxel and Sintilimab was initiated. Repeat CT in February 2023 demonstrated a partial response with 62% reduction in the lung mass. Durable disease control has been maintained through May 2023, with overall survival of 18 months so far (**Figure 1**).

## Discussion

SCLC is an aggressive neuroendocrine malignancy representing 14% of lung cancers (1). The high proliferative rate and early metastatic potential lead to dismal prognosis, with 5-year survival around 7% (10). First-line platinum doublets result in high response rates up to 70%, but nearly all SCLC patients relapse quickly (3). The median overall survival with first-line chemotherapy is only 10 months (4). Common second-line options include topotecan, amrubicin, and lurbinectedin, but efficacy is modest and short-lived (5, 11). By the third-line setting, treatment choices are limited and outcomes remain poor.

Recent Advances with Immunotherapy

ICIs targeting the PD-1/PD-L1 axis have shown promising clinical activity in SCLC and NSCLC, which are now approved as first-line and later-line therapies (6–8). Despite this progress, only a minority of SCLC patients respond to ICIs. Proposed reasons include lack of TILs, immunosuppressive tumor microenvironment, and suboptimal patient selection (5, 8). Identifying robust predictive biomarkers represents a key unmet need in this disease.

**Figure 2:**
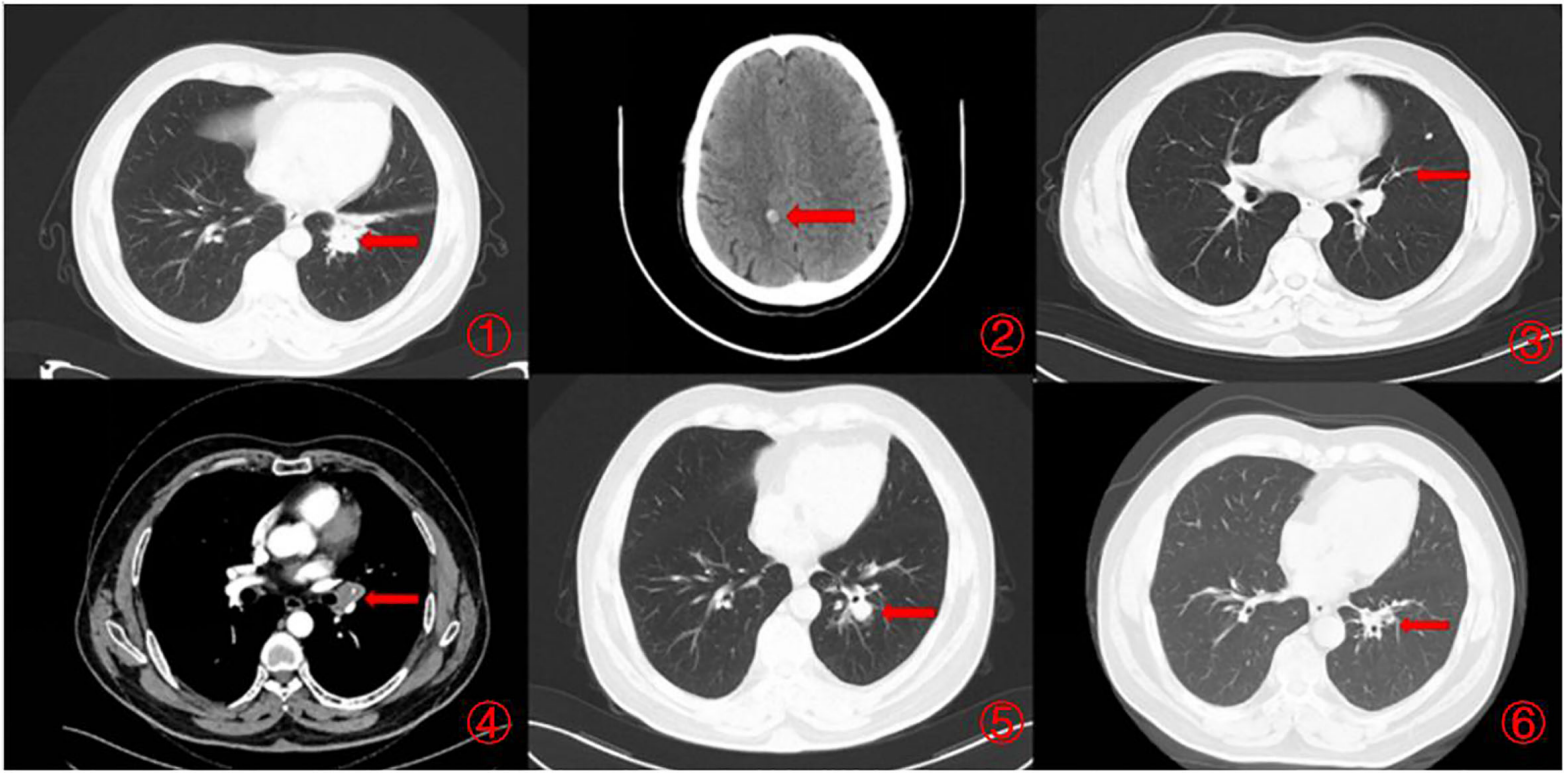
Imaging changes during treatment. CT images demonstrating diagnosis, treatment responses, and progression. [(1, 2) Small cell lung cancer was first diagnosed (December 2021), CT scan showed a 3.8×2.2cm pulmonary nodule and brain metastases. (3). Minimal lung lesions during first-line chemotherapy (February 2022), CT scan showed a 0.8×1.4cm pulmonary nodule. (4). Progression after completion of first-line chemotherapy (May 2022), CT scan showed a 1.3×1.7cm pulmonary nodule. (5). Progression after completion of second-line chemotherapy (September 2022), CT scan showed a 2.1×1.6cm pulmonary nodule. (6). Partial remission was achieved after immunotherapy (February 2023), CT scan showed a 0.9×0.5cm pulmonary nodule).

Our case demonstrates exceptional and durable response to Sintilimab in the third-line setting, with 62% tumor reduction by RECIST and progression-free survival of 9 months so far (**Figure 2**). However, exceptional responders like this patient remain the exception rather than the norm. Ongoing research is focused on better understanding and enhancing antitumor immune responses in SCLC. Efforts are underway to develop more rational biomarker-driven combination strategies to extend the benefits of immunotherapy to a greater proportion of SCLC patients,Previous studie have achieved favorable treatment outcomes with similar treatment regimens (7, 12).

### Potential predictive biomarkers

In our case, the patient’s NLR and CEA levels closely paralleled his response to Sintilimab (**Tables 1**, **2**). NLR peaked before immunotherapy then rapidly declined, mirroring his tumor regression. Elevated NLR is associated with poor prognosis across cancer types (13, 14). The sharp drop in NLR following immunotherapy corresponds with the patient’s exceptional outcome. Blood-based tumor mutational burden has also shown early promise as a predictive marker for ICIs in relapsed SCLC (12, 15). These findings suggest CEA and NLR warrant further study as potential biomarkers of ICI benefit in SCLC.

**Table 1:** CEA levels during treatment.

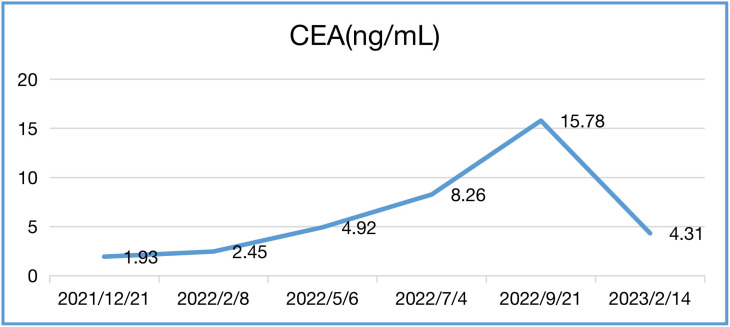

**Table 2:** NLR during treatment.

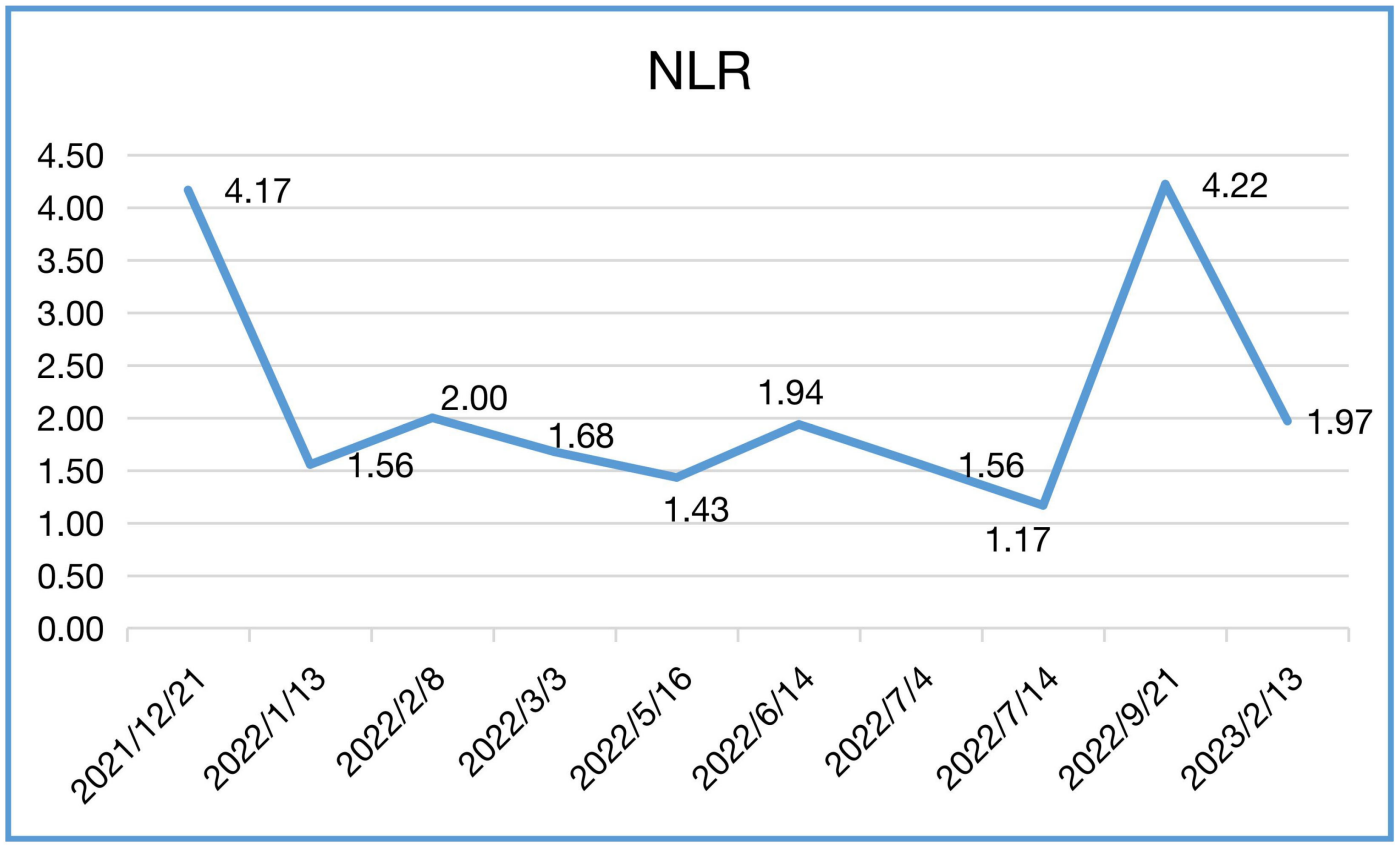

## Conclusions

In summary, our case demonstrates remarkable antitumor activity of the PD-1 inhibitor Sintilimab in a heavily pretreated SCLC patient. ICIs represent a promising new therapeutic avenue for some SCLC patients who have exhausted standard therapies. Ongoing research aims to expand the proportion of SCLC patients deriving durable benefit from immunotherapy through development of reliable predictive biomarkers and rational combination approaches.

## Data availability statement

The original contributions presented in the study are included in the article/supplementary material. Further inquiries can be directed to the corresponding author.

## Ethics statement

The studies involving humans were approved by Ethics Committee of the First Hospital of Jilin University. The studies were conducted in accordance with the local legislation and institutional requirements. The participants provided their written informed consent to participate in this study. Written informed consent was obtained from the individual(s) for the publication of any potentially identifiable images or data included in this article.

## Author contributions

GY: Writing – original draft. XL: Writing – review & editing. XZ: Writing – review & editing, Data curation. WS: Writing – review & editing, Investigation. JL: Writing – review & editing, Project administration. ZD: Writing – review & editing. XC: Supervision, Writing – review & editing.
